# Experiences of Rehabilitation Professionals with the Implementation of a Back School for Patients with Chronic Low Back Pain: A Qualitative Study

**DOI:** 10.1155/2016/6720783

**Published:** 2016-07-03

**Authors:** Stefan Peters, Hermann Faller, Klaus Pfeifer, Karin Meng

**Affiliations:** ^1^Department of Medical Psychology, Medical Sociology and Rehabilitation Sciences, University of Würzburg, Klinikstraße 3, 97070 Würzburg, Germany; ^2^Institute of Sport Science and Sport, Chair of Exercise and Health, Friedrich-Alexander University Erlangen-Nürnberg, Gebbertstraße 123b, 91058 Erlangen, Germany

## Abstract

A standardized curriculum back school (CBS) has been recommended for further dissemination in medical rehabilitation in Germany. However, implementation of self-management education programs into practice is challenging. In low back pain care, individual factors of professionals could be decisive regarding implementation fidelity. The study aim was to explore attitudes and experiences of professionals who conducted the back school. Qualitative interviews were led with 45 rehabilitation professionals. The data were examined using thematic analysis. Three central themes were identified: (a) “back school as a common thread,” (b) “theory versus practice,” and (c) “participation and patient-centeredness.” The CBS and its manual were frequently described positively because they provide structure. However, specified time was mentioned critically and there were heterogeneous perceptions regarding flexibility in conducting the CBS. Theory and practice in the CBS were discussed concerning amount, distribution, and conjunction. Participation and patient-centeredness were mainly mentioned in terms of amount and heterogeneity of participation as well as the demand for competences of professionals. Factors were detected that may either positively or negatively influence the implementation fidelity of self-management education programs. The results are explorative and provide potential explanatory mechanisms for behavior and acceptance of rehabilitation professionals regarding the implementation of biopsychosocial back schools.

## 1. Introduction

Self-management education is a central part of medical rehabilitation and has been proven effective in many clinical populations regarding a variety of outcomes [1–3]. Throughout recent decades, the characteristics of self-management education programs have changed. Biopsychosocial [4] and interprofessional [5] principles have been incorporated and the paradigms of empowerment [6] and patient-centeredness [7] have profoundly affected the methods of delivery and led to a more interactive approach [1]. Quality requirements such as the use of manuals and patient-oriented didactics were widely established for self-management education programs (e.g., [8, 9]).

Programs for persons with low back pain are a prominent example for the above-mentioned changes. In the early 1990s, the so-called back schools rarely incorporated psychosocial factors [10], whereas nowadays these factors are regarded as paramount in this population [11–14]. Secondly, the patient-centered relationship of healthcare professional and patient can best be described, as Slade et al. ([15, p999]) have put it, “consultative, rather than prescriptive,” prioritizing patients' preferences and individual circumstances.

A standardized curriculum back school (CBS), built upon the outlined biopsychosocial and patient-centered principles, has proven to be effective within inpatient medical rehabilitation regarding outcomes such as illness knowledge, coping with pain and conducting home exercises [16]. The program consists of 7 sessions/modules with 60 minutes each and is interdisciplinary, with both physicians and psychologists delivering one session each and physiotherapists/exercise therapists five sessions, respectively. A theory-driven approach is used to foster long-term adherence to physical activity by systematic inclusion of behavior change techniques [17]. The CBS manual outlines contents as well as methods in a detailed manner. A further dissemination of the CBS in inpatient medical rehabilitation was recommended.

However, implementation of innovations in healthcare is challenging [18] with influencing factors present on several levels, such as the individual professional, the organization, and the political context [19]. In Germany, implementation studies of self-management education programs [20, 21] have brought to light various barriers and facilitators. The former included a lack of adequate staff, problems with therapy planning (e.g., time management), and external factors such as an unsteady allocation of patients. Facilitators were, for example, positive feedback from patients as well as earlier experiences with self-management education programs and a high motivation of rehabilitation professionals. Of all influencing factors, organizational variables were most precisely measured, whereas individual factors of professionals played only a subordinate role. In the field of low back pain management, a particularly difficult area for implementation of innovations [22], individual factors could be decisive though. The biopsychosocial paradigm shift has not always reached the scope of therapists and is at least challenging for them or they are prone to a more mechanical perspective [12, 23, 24]. This might negatively influence “implementation fidelity” of innovations such as the CBS, that is, “(…) the degree to which (…) providers implement programs as intended by the program developers” ([25, p240]). This on the other hand poses a threat to the effectiveness of the CBS in routine care.

In a larger trial in initially 10 inpatient rehabilitation facilities in Germany, the CBS had been implemented into routine care [26]. This was carried out with 2 different implementation interventions, a written guideline and a train-the-trainer workshop. The aim was a comparison of guideline and workshop in terms of the implementation fidelity of the back school. All clinics reached at least an acceptable rate of fidelity but modifications to the back school were common and varied greatly [27]. To account for influences on implementation fidelity, an embedded qualitative study was conducted after the CBS had been implemented. The aim was to explore attitudes and experiences of rehabilitation professionals who conducted the CBS with the purpose of identifying central factors that might be barriers or facilitators to implementation fidelity.

## 2. Methods

Ethical approval for this study was obtained from the respective commission of the medical faculty of the University of Würzburg, Germany (decision on the 25 March 2011). The Consolidated Criteria for Reporting Qualitative Research statement (COREQ) was used as guidance for this paper to ensure transparency [28].

### 2.1. Data Collection

Twelve weeks after the implementation of the CBS had been initiated, individual interviews were conducted with 45 rehabilitation professionals (27 women) from 9 rehabilitation facilities (1 clinic had decided not to implement the back school): 9 physicians, 10 psychologists, 17 physiotherapists, 6 exercise therapists, and 3 occupational therapists. Their mean age was 37.4 (SD = 9.3). They were selected by the respective clinics and between 4 and 8 professionals were interviewed from each clinic. 27 of all current study participants had already taken part in interviews prior to the program implementation [29]. It is not known to the authors, whether approached interviewees refused to participate. An interview guide was used, comprising main topics and follow-up questions (see “*Interview Guide with Follow-Up Questions*”).

*Interview Guide with Follow-Up Questions*

- What is your opinion concerning the curriculum back school (CBS), now that it has been implemented at your clinic?
  - Are there aspects in the CBS that you do not agree with?
- What was your specific role in the implementation of the CBS?
- Which experiences did you gather in the organizational implementation of the CBS at the clinic?
  - Which factors complicated the implementation?
  - Which factors facilitated the implementation?
  - When difficulties occurred: What did you do against it? Respectively, what do you plan to do against it?
- Which experiences did you gather when conducting the back school?
  - Which factors complicated the execution?
  - Which factors facilitated the execution?
  - When difficulties occurred: What did you do against it? Respectively, what do you plan to do against it?
- What were the consequences of the implementation of the CBS?
  - regarding you personally
  - regarding colleagues
  - regarding the interdisciplinary collaboration
  - regarding the clinic
  - regarding patients
  - positive/negative?

(vi) How do you think things will continue with the CBS at your clinic?

31 interviews were led by the first author (Stefan Peters), a male exercise scientist, and 14 interviews were led by a female psychologist (Anja Schultze, see Acknowledgments), both with prior experiences in conducting and analyzing qualitative interviews [29]. They worked as researchers at the Universities of Erlangen-Nürnberg and Würzburg, respectively, at the time and had been in contact with most interviewees earlier in the study. The interviews lasted on average 29 minutes (SD = 9.5) and took place at the rehabilitation clinics. Except participants and researchers, nobody else was present. All study participants provided consent regarding audiotaping and transcription of their interviews. Sensitive data of all kind were rendered anonymous in the process of transcription. Each participant gave permission for his interview data to be evaluated.

### 2.2. Data Analysis

The transcripts were analyzed with the software Atlas.ti [30] using thematic analysis [31]. For coding, main categories were derived from the implementation model of Grol and Wensing [19]: “Innovation,” “individual professional,” “patient,” “social context,” “organizational context,” and “economic and political context.” In this study, the innovation was the implemented CBS and the individual professional was the respective interviewee in his or her role as trainer. Matching of statements with the above-mentioned categories was defined as selection criterion (other statements were not included in the analysis). In a first step of the analysis, the first and the last author (Stefan Peters, Karin Meng), both from different professions (Karin Meng as psychologist), read a large part of the textual corpus to become familiar with the data. Secondly, all “meaning units,” that is, “a segment of text that is comprehensible by itself and contains one idea, episode or piece of information” ([32, p116]), were coded inductively by both researchers. In a third step, one coder (Stefan Peters) identified preliminary themes. This was carried out by searching for similarities and overlaps between codes and for patterns in the data, respectively [33]. Themes were regarded as pattern-like or central, if they were repeated both in the individual interviews and across different clinics and professions [34]. An additional indicator was that themes contain various aspects but follow a central organizing concept [31, 33]. In an iterative process, preliminary themes were compared with individual codes and final central themes were named and defined. The analysis was carried out from an essentialist/realist point of view and aimed at reporting on the experiences and the reality of the participants as they display it [31]. A semantic approach, which takes into account the “explicit or surface meanings of the data” ([31, p84]), was therefore used for coding and theme creation.

For this paper, all quotations as well as the interview guide (see “*Interview Guide with Follow-Up Questions*”) were translated from German into English by the first author (Stefan Peters).

## 3. Results

**Figure 1:**
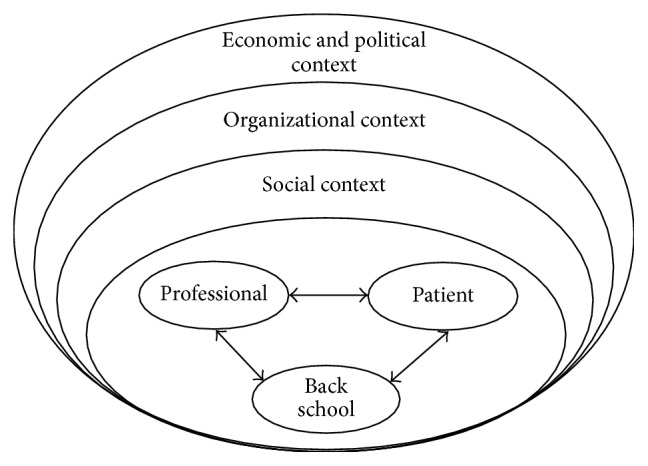
Levels of implementation as reflected in this study, as adapted from Grol and Wensing [19].

While themes regarding the various contexts (social, organizational, economic, and political) were mentioned less frequently, the interviewees talked more about the CBS (“innovation”), the “patients,” and themselves (“individual professional”). These three levels displayed a pronounced conjunction (e.g., many interviewees described attributes of the back school via views and expectations of patients) (Figure 1).

**Figure 2:**
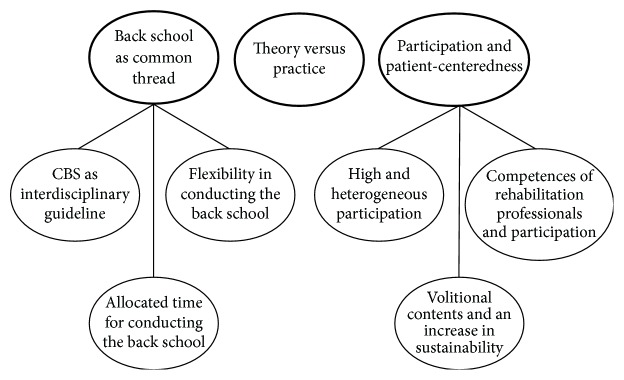
Central themes derived from the analysis.

Across these levels, three central themes were identified: (a) “back school as common thread,” (b) “theory versus practice,” and (c) “participation and patient-centeredness.” For (a) and (c), several subcategories were identified (Figure 2). An in-depth description of all themes and subcategories is presented below.

### 3.1. Back School as Common Thread

#### 3.1.1. Curriculum Back School as (Interdisciplinary) Guideline

Many interviewees described the CBS or its manual, respectively, as “common thread.” For them, that was something which creates a “systematization regarding presentation” or an “entirely clear structure” or is “systematic” or a “plan.” The connotation of this notion was mostly positive.

Physician:* “(…) it is surely good to be provided with a concept. The curriculum consists of 7 modules, those are the contents! That was surely valuable (…). That certainly helped us a lot, because we did not have to consider, what we need for it, but: ah, it's written here.”*

Thereby, the rehabilitation professionals highlighted the interdisciplinary character of the back school. They stressed that this creates a “connection” between contents and overcomes “different approaches.”

Psychologist:* “Well, that I have the feeling that we do, that we as clinic act in concert now (…). That one department does not do one thing, works with other terms and we again do something different with different terms. So I just regard the clarity for patients as higher.”*

Some interviewees explicitly outlined media and material, which accompany the back school as structure providing.

Exercise therapist:* “All of that was made easier for me by your slides, yes, I mean I have slides of my own as well and that way, that way one had a strict plan, also with those, what I liked, the flipcharts (…). That is clearly arranged.”*

#### 3.1.2. Specified Time for Conducting the Back School

However, the specified time for certain contents in the “common thread” back school was critically mentioned. Across clinics and professions, a lack of time was described when conducting the back school.

Physician:* “The situation here is that the physician lecture or the physician seminar are very comprehensive in terms of contents. It partly goes too much into detail (…). In every module 1, we came into conflict with the time. It was never completed with all slides having been presented (…).”*

Obviously, the lack of time did not merely come from the extent of contents but also from participation of patients and the use of interactive methods, respectively.

Psychologist:* “Time pressure. Well, I regard this as very problematic: not enough time at all. Whenever one suddenly starts to discuss things, time has already passed again; one has to think about the next thing. One cannot simply let things slide if you want to impart the content.”*

The lack of time led to a variety of implications for the interviewees. Some described that they were able to correct exercises only to a lesser extent; others had left out contents or dealt with contents or methods flexibly.

In some cases, the time that is provided by the new back school was characterized positively in comparison with the traditional back school that had been in place at the clinic previously.

Exercise therapist:* “(…) it is more fun for me; I have more time at hand than in our conventional back school. Personally, I find this very comfortable.”*

#### 3.1.3. Flexibility in Conducting the Back School

The flexibility in dealing with the “common thread” back school was another topic across clinics and professions. A supposedly strict “one-to-one” adoption of the curriculum back school seems to have led to difficulties or a rather negative perception of the “guideline.”

Occupational therapist:* “That's a bit like a sword behind your back, where one thinks: Hmm, now you have to see that you pull it through, that you just also stick to a common thread. And that is not always easy to establish if you work together with emotionally reacting human beings.”*

Handling the CBS manual flexibly appears to have made it easier to conduct the program and promoted the satisfaction of rehabilitation professionals.

Physiotherapist:* “(…) there I have a common thread, but I can step out of line at both left and right. I regard this as very easy to implement.”*

The perception of how flexible or not one can handle the back school program seems to be rather stable within individuals and appears to have persisted, even if it was well known that a certain variability is possible.

Physiotherapist:* “And that everything so exact, precisely accurate, 10 minutes for this issue, 10 minutes for that or 5 minutes for that issue. I think of this as a little bit, that is bound too strictly. However, you have said it already, one is a little bit flexible (…).”*

It became apparent that the use of the manual requires certain competences.

Physiotherapist:* “There I just realized that different kinds of therapists exist. Some like giving lectures and also teach at physio-schools, for them it is easy to prepare such a lesson. They take that thing [manual of the back school], flip through it and in their head, a structure starts to emerge as to how they conduct the class later. Others just struggled a little bit more to acquaint themselves (…). Well, they just oriented themselves very accurately towards the concept [manual of the back school] and just think “I now conduct the introduction for 7 minutes and then I may speak 5 minutes about this and then comes 12 minutes about that und what shall I do if I need 15 minutes there instead of 12?” Well, simply a little bit too strict.”*

### 3.2. Theory versus Practice

Interviewees across clinics and professions addressed amount, distribution, and conjunction of theory and practice in the back school program. It was often stated that patients wanted to have more practice and less theoretical content. This relates, for example, to the patients criticizing too much sitting.

Physiotherapist:* “One did also see that all of them then became a little bit more restless, the patients. (…) starting with the 3rd, 4th session [of the back school], not later than with me in the 5th, or so, that they became rather restless and simply said: “Ha, today it's theory again, only sitting again” (…) because it is really theory-loaded and listening, receiving so much theory.”*

From their own point of view, the interviewees also described their wish for more practical content or explained that this comes more naturally to them. In part, they illustrated this by reference to their professional role.

Physiotherapist:* “(…) to teach those didactics to the patient. So, you have to make a plan for yourself and this is how a plan may look like [content of action and coping planning in the CBS]. That is more what perhaps exercise therapists do or psychologists. Well, we physiotherapists work more practically.”*

Theory was also addressed concerning its transfer into practice or everyday life.

Psychologist:* “(…) it surely is very theoretical (…) that is certainly not realizable at all (…). And this transfer that they should do, (…) this was not yet in my line.”*

Ambivalences regarding the amount of theory became evident as well.

Exercise therapist:* “(…) session 6 and 7, that is when one just sits idle again, but exactly to talk about that, what's it like at home, I regard this as very, very good, to include this.”*

Several interviewees connoted the conjunction of theory and practice positively.

Physiotherapist:* “Well, in the beginning, this touching on one's own body [e.g., touching shoulder blade or bodies of vertebra], that is what (…), what establishes the connection between theory and practice. Well, that is very meaningful. Well, that is something, which we did not do previously for example, and where one simply notices that the participants are really excited and really simply feel this and yes, experience themselves in this.”*

### 3.3. Participation and Patient-Centeredness

#### 3.3.1. High and Heterogeneous Participation

The participation of patients was mostly described as high, which was partly portrayed as a difference in comparison to the former back school program.

Physician:* “Well, I have fun with that and one recognizes that the patients are interested and that they participate and that they are vigilant and then I think, one can perhaps make a difference (…) that after all something happens at home.”*

It was surprising for many interviewees that participation was so high.

Physiotherapist:* “I have thought: Oh dear, if I think about our patients, will they participate? But probably it [previously] had been the case that they sat in the group, let us call it lethargically, because I guess you yourself had chosen only the teacher-centered teaching, in inverted commas.”*

On the other hand, the rehabilitation professionals often recognized a certain heterogeneity in patients regarding their participation. They mostly saw the reasons for this in varying motivation and acceptance of some patients.

Psychologist:* “There were simply those who understood quickly, who innately had little reservations. They participated actively and contributed well. However, there simply were many who, in that case, did not participate actively.”*

#### 3.3.2. Competences of Rehabilitation Professionals and Participation

Concerning the competences with regard to the interactive group facilitation, there was an obvious heterogeneity among professionals.

Exercise therapist:* “(…) that you worked a lot with questions and let people feel something. However, I know it this way, because I already completed the back school license in the new back school and there it is the usual way to include people more. (…) while our three physiotherapists were a bit critical in the beginning, perhaps a bit insecure. One of them is a bit older. (…) he simply knows that, the original form so to say, and this teacher-centered teaching. And this certainly influences you and there one realized that he is a little bit insecure in the beginning, how to conduct it all [the back school program], struggles a little bit (…).”*

It became apparent that an increased patient participation or the empowerment of patients, respectively, could supposedly make it easier for rehabilitation professionals to conduct the back school.

Physiotherapist:* “(…) I mean for me it is simply an alleviation actually. Now it is not the case anymore that everybody thinks: Yes, she tells me now how to do it right actually, but they exercise on their own and there is no simple solution, that you click your fingers, but they simply have to give thought to it themselves: What could I do, what could I do better or what could I do more often (…)? As such, the pressure is somewhat, actually, not on me anymore.”*

#### 3.3.3. Volitional Contents and an Increase in Sustainability

The volitional contents of the back school (behavior change techniques such as action planning, barrier management, and self-monitoring) met the approval of most interviewees. On the one hand, they were characterized as theory-loaded (see theme “Theory versus Practice”), but they were regarded as substantial to support the transfer into everyday life. This approval was even expressed by rehabilitation professionals for whom these contents were new or who had difficulties conducting them.

Physiotherapist:* “(…) what was really new for me was the barrier management. That is previously, when I conducted this, I didn't go into that. But now I go into that because this is actually indeed the biggest problem that people have, implementing sporting activity in everyday life, because there certainly emerges one's weaker self and stress and so on.”*

## 4. Discussion

This study explored potential barriers and facilitators regarding the implementation fidelity of the CBS, which had been implemented in 9 rehabilitation facilities. For this purpose, the rehabilitation professionals who conducted the program were interviewed about their attitudes and experiences with regard to the program.

The first finding concerns the importance of individual factors. Central themes mentioned by the interviewees were located on a conjunction of the levels “innovation,” “individual professional,” and “patient” as described in the implementation model of Grol and Wensing [19]: “back school as common thread,” “theory versus practice,” and “participation and patient-centeredness.” Regarding these themes as well as their respective subcategories, barriers and facilitators could be located.

Rehabilitation professionals mostly evaluated the alleged characteristic of the CBS as a common thread, guideline, or structure-providing framework as positive. This holds true in particular for the interdisciplinary structure or the common language provided. The role of structure and guidance and its mainly positive perception by rehabilitation professionals had already been pointed out in interviews prior to the implementation of the CBS [29]. In contrast, barriers could be located in two subcategories. Very often, the interviewees reported difficulties in conducting the content of the back school in the time dedicated for the respective contents. Additionally, varying degrees of flexibility in conducting the back school with regard to the manual's demands emerged. A deviation from the manual might be seen as a threat to implementation fidelity. However, interventions usually have core and noncore components with the latter being open to a certain degree of modification [35]. When the CBS was implemented, it was outlined that specific content did not have to be delivered par for par (e.g., examples of how to say something literally) and can be tailored to the group (e.g., adaptation of exercises with regard to the performance level). In interviews that were precise enough concerning this topic, professionals indicated in part a very strict perception of the back school program, which went as far as to a literal use of the example quotations. It remains open to speculation though, as to how the individual professionals define “flexibility.” Rutten et al. [36] claim low flexibility as one of the important determinants of lacking guideline-adherence among physiotherapists in the treatment of low back pain. Possibly, professionals might perceive too much strictness as constraint of their professional autonomy (cf. [37, 38]). When drawing inferences from research about guidelines, however, specifications of how to carry out a certain behavior are paramount. Michie and Johnston [39] have pointed out that specific instructions make it more likely that guidelines are put in place. However, self-management education programs in medical rehabilitation in Germany often lack a manual [40], which might indicate that a majority of rehabilitation professionals are not experienced in working with a manualized program. Short and simple implementation interventions such as written educational material might therefore be insufficient for professionals to gain confidence in working with a specific manual. Regarding content of the manual, it seems important that program developers clearly point out where flexibility is possible or even advocated.

Lack of experience with handling a manual could also be the reason for the difficulties the rehabilitation professionals had with delivering the respective content of the CBS within the dedicated amount of time. In the interviews, however, these difficulties were reported so frequently that multiple reasons seem plausible. Interactive methods and patient participation often appeared to be challenging for the rehabilitation professionals, because this seemingly required a lot of time. Additionally, the back school content was, to some extent, portrayed as quite complex and a certain introduction, for example, for psychological topics, was regarded as necessary. One psychologist mentioned that his usual pain course consists of several sessions with a duration of 90 minutes, which is more time than he has in the psychological session in the CBS. Another session of the CBS covers back posture and the so-called “back-friendly behavior” in everyday life. Rehabilitation professionals might have perceived that less time was available for a content that had been very prominent in their conventional back schools according to findings from interviews and selective visitations prior to the implementation of the CBS [29, 41].

The comparison with the traditional back school program also seems to be the reason why rehabilitation professionals frequently alluded to the ratio between theory and practice in the CBS. Not surprisingly, the CBS was characterized as having more theory than the traditional programs of the clinics. The latter had mostly not been interdisciplinary, had focused more on back posture and physical exercises, and had not contained sessions explicitly aiming at motivation and volition to foster long-term physical activity or other psychosocial contents [41]. A majority of interviewees connoted the allegedly high amount of theory in the new program negatively. Very occasionally, the statements provided a hint that the negative appraisal by patients of the amount of theory or sitting instead of exercising resulted from certain implementation processes (e.g., patients received certain contents repeatedly during the rehabilitation treatment). This was the case when a physician lecture for low back pain patients contained similar content as the session of the physician in the CBS and was kept in place even though the CBS had been implemented. Some rehabilitation professionals referred to their professional role when discussing the amount of theory and practice. From a traditional paradigm, low back pain is often approached as a mechanical problem or disorder [42, 43]. This leads to respective treatment options that target, for example, strength or mobility or movement patterns (e.g., [24]) and might thus be perceived as more practical than psychosocial approaches. On the other hand, behavior change content of the back school was valued by professionals as illustrated in the theme “volitional contents and an increase in sustainability.” Recent systematic reviews highlighted that a significant proportion of musculoskeletal physiotherapists perceive psychological interventions as normal part of their practice [44] but seem to have difficulties in conducting the interventions due to a lack of training [24, 44]. The theory-based behavior change content might therefore be one reason for the ambivalence of rehabilitation professionals regarding the amount of theory and practice that became clear in this study. To improve acceptance among professionals, slight modifications of the CBS to change the theory/practice mix within class sessions might also be worth considering on a case-by-case basis. Indeed, for complex interventions, a “specified degree of adaptation to local settings” seems to be appropriate [45]. However, it should be kept in mind that the stronger the adaptations are, the more the effectiveness of the program in routine care has to be called into question.

Patient participation in the CBS was mostly described as high. In another study in medical rehabilitation in Germany, patients had shown a high appreciation for being encouraged to participate more strongly during educational courses by rehabilitation professionals. They had indicated that it made it easier for them to follow and understand the content of self-management education programs [46]. Still, the interviewees in the current study also mentioned a considerable heterogeneity in terms of patient participation. Next to patients' varying degrees of acceptance and motivation, the individual competences of professionals could play a role, as they were also labelled as heterogeneous. Not surprisingly, rehabilitation professionals connoted the high participation by patients very positively. They had already indicated prior to the implementation of the CBS that sustainability was one of their expectations and they associated this goal with patient participation [29]. A recent review stated that patients' adherence is fostered by a positive “working alliance between provider and patient” ([47, p43]).

It is noticeable that issues regarding the implementation intervention (implementation guideline, train-the-trainer workshop) were barely mentioned. However, a differentiation between clinics which implemented the CBS via either guideline or workshop had not been the research question in this interview study. Therefore, the interviews contained no explicit question regarding that issue.

There are several limitations of this study, which have to be taken into account. The self-selection of both the rehabilitation clinics in general as well as the interviewees in particular might limit the external validity. The authors do not know whether there were rehabilitation professionals who refused to be interviewed because the clinics were in charge of selecting the interviewees. While nine rehabilitation clinics can be considered a high number of facilities for implementation studies, it might still have been a positive selection since there was no random drawing. However, when including clinics into the study, a variability in terms of sponsor/owner and characteristics of patients (e.g., amount of patients with chronic low back pain) was taken into account. Additionally, when interpreting the results, the characteristics of the study sample should be considered. The sample consisted of trainers, while administration staff and other stakeholders at the clinics were not interviewed because of the research question. This might be one explanation why individual factors were mentioned more commonly as compared to organizational factors. Furthermore, the thematic analysis that was used did not aim at a summary of all the data but at the identification of central themes that came up in the interviews. There was no explicit cut-off for a theme to be “central,” and the themes are not without a certain overlap.

## 5. Conclusion

This study detected factors that may either positively or negatively influence the implementation fidelity of self-management education programs. The central themes of this interview study covered a conjunction of the implementation levels “innovation,” “patient,” and “individual professional.” However, the results are explorative and hypothesis-generating and provide potential explanatory mechanisms for behavior and acceptance of rehabilitation professionals concerning the implementation of the CBS. Regarding this topic, data are scarce for German medical rehabilitation in general or the self-management education in this setting in particular. Educational needs of rehabilitation professionals conducting self-management education programs should be further explored in future research. A crucial future research question may ask what measures may support them bringing the use of manuals in line with their individual practice patterns and a varying degree of patient participation. Particularly the issues of flexibility and time management appear to be important, together with trainer competences for conducting patient-centered, interactive groups. Those are also important topics for educational offers, supporting rehabilitation professionals. Such offers are likely to enhance implementation fidelity of standardized, patient-centered self-management education programs like the CBS.
